# An Investigation into Tetrodotoxin (TTX) Levels Associated with the Red Dorsal Spots in Eastern Newt (*Notophthalmus viridescens*) Efts and Adults

**DOI:** 10.1155/2018/9196865

**Published:** 2018-09-02

**Authors:** Mackenzie M. Spicer, Amber N. Stokes, Trevor L. Chapman, Edmund D. Brodie, Edmund D. Brodie, Brian G. Gall

**Affiliations:** ^1^Department of Pharmacology, The University of Iowa, Iowa City, Iowa 52242, USA; ^2^Department of Biology, California State University, Bakersfield, CA 93311, USA; ^3^Department of Biology, East Tennessee State University, Johnson City, TN 37614, USA; ^4^Department of Biology, Utah State University, 5305 Old Main Hill, Logan, UT 84322, USA; ^5^Department of Biology, University of Virginia, PO Box 400328, Charlottesville, VA 22904, USA; ^6^Department of Biology, Hanover College, 517 Ball Drive, Hanover, IN 47243, USA

## Abstract

We investigated the concentration of tetrodotoxin (TTX) in sections of skin containing and lacking red dorsal spots in both Eastern newt (*Notophthalmus viridescens*) efts and adults. Several other species, such as* Pleurodeles waltl* and* Echinotriton andersoni,* have granular glands concentrated in brightly pigmented regions on the dorsum, and thus we hypothesized that the red dorsal spots of Eastern newts may also possess higher levels of TTX than the surrounding skin. We found no difference between the concentrations of TTX in the red spots as compared to neighboring skin lacking these spots in either efts or adults. However, efts with more red dorsal spots had elevated TTX levels relative to efts with fewer spots.

## 1. Introduction

Tetrodotoxin (TTX) is a nonproteinaceous neurotoxin that prevents the generation of action potentials in nerve and skeletal muscle by binding to the pore region of voltage-gated sodium channels, leading to fatal asphyxiation [1, 2]. This natural toxin is found in a wide range of taxa including marine bacteria, ribbon and flatworms, mollusks, and several species of terrestrial amphibians (reviewed by [3–5]). One of the primary functions of TTX is as an antipredator defense mechanism. This ecological role has been well documented in rough-skinned newts (*Taricha granulosa*), in which high concentrations of TTX are found in the dorsal skin of adults [6]. When attacked, salamanders compress myoepithelial sheaths that surround granular glands in the skin [7]. This forces the toxin out of the gland, which may then be absorbed by epithelial cells in the predator's mouth, leading to rapid death in nonresistant predators [8].

While newts from the western US possess the highest concentrations of TTX, the Eastern newt (*Notophthalmus viridescens*) also possesses this potent toxin [9]. In the United States, Eastern newts range from Maine to Florida and west to Texas [10]. The adults are fully aquatic and contain relatively low concentrations of TTX [9, 11]. However, the juvenile stage, known as the red eft, is terrestrial and contains roughly ten times higher concentrations of TTX that correspond to their bright orange aposematic coloration ([9, 11]; but see [12, 13]). Like newts from the western US, the TTX present in the skin of Eastern newts is able to help deter some predators [11, 14, 15]. Eastern newts are characterized by their red, dorsal spots [10]. These spots vary in brightness and number [16] and could potentially be areas with concentrated granular glands. Other newts are also known to contain regions of skin with high concentrations of toxin. For example, both* Pleurodeles waltl* and* Echinotriton andersoni *[17, 18] possess lateral ribs that penetrate the epidermis when attacked [17, 19, 20]. As the ribs protrude from the skin, they pierce areas with concentrated granular glands, which coat the sharp tip of the rib in toxin, leading to its injection into the predator's mouth [17, 21]. Since Eastern newts exhibit tremendous variation in the brightness and number of spots [16], we hypothesized that these spots could possess higher concentrations of TTX relative to the surrounding skin. Additionally, the relationship between the number of red dorsal spots and toxicity is unknown. Thus, we assayed the levels of TTX from skin containing red dorsal spots and skin lacking these spots in adult and eft newts. In addition, we counted the number of red dorsal spots on each individual eft and compared it to the predicted whole body toxicity.

## 2. Materials and Methods

Eastern newts were collected from Mountain Lake Biological Station, Pembroke, Virginia, in 2015 under Virginia Scientific Collection Permit #054260. A total of seven adult newts (mean ± SD: 2.08±0.32 g) and 15 efts (1.82±0.71 g) were collected. Animals were anesthetized via immersion in 1% tricaine mesylate (MS222) buffered to a pH of 7. The number of red dorsal spots was counted (efts only) and skin punches were taken using 2.0 mm and 3.0 mm punches for the eft and adult newts, respectively. A single red spot was then haphazardly selected on each animal and removed. A section of skin lacking a red spot was also removed from the opposite side of the animal. Samples were immediately frozen (-80°C) in individual microcentrifuge tubes until TTX extraction and quantification. After removal of skin punches, newts were euthanized in a 10% solution of MS222 and frozen. Extraction of skin punches was performed according to Hanifin et al. [6], and quantification was executed using a Competitive Inhibition Enzymatic Immunoassay (CIEIA) as in Stokes et al. [22]. This assay is highly specific and works by binding anti-TTX monoclonal antibodies to TTX. In the absence of TTX or in low concentrations of TTX, the antibodies bind to the conjugate on the plate allowing secondary antibodies to also bind to the plate, resulting in a high absorbance reading. This value is then used to calculate the TTX concentration using a linear standard curve. The assay is able to detect TTX at a minimum concentration of 10 ng/mL and has a linear range of 10–500 ng/mL [22]. All plates were read at 405 nm.

We compared the amount of TTX per skin punch from dorsal patches of skin containing and lacking red spots from adult and eft newts with paired t-tests (Sigmaplot 12.5, Systat Software Inc., San Jose, CA). To compare eft and adult TTX, we multiplied the amount of TTX in each punch from an adult newt by a factor of 0.556 or the difference in surface area (mm^2^) between a 3 mm and 2 mm skin punch. We then calculated the average toxicity for each individual (combining the punch with and without a red spot) and compared adult and eft toxicity using a t-test. Finally, using this average we also calculated the average TTX per plug for the spotted and unspotted patches of skin and estimated whole eft TTX using the methods of Hanifin et al. [23]. We then compared predicted whole newt TTX to the number of red dorsal spots using linear regression (efts only).

## 3. Results

**Figure 1:**
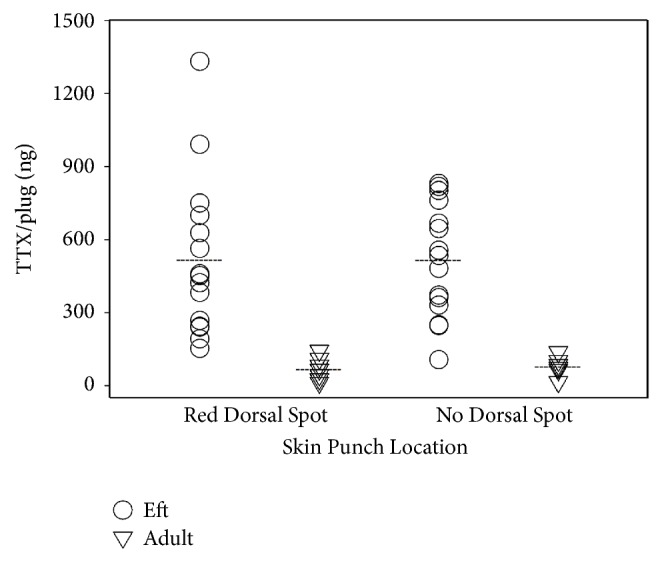
Concentration of tetrodotoxin (TTX) per plug (ng) in brightly spotted and unspotted dorsal epidermis samples from Eastern newt (*Notophthalmus viridescens*) efts (circles) and adults (triangles). A paired t-test found no significant difference between the mean concentrations of TTX in pigmented samples (*t* = 0.014; df = 14;* P* = 0.989) from efts or adults (*t* = -0.453; df = 6;* P* = 0.667). Means are represented by dashed lines. Adult newts have significantly lower concentrations of TTX in their skin than efts (*t* = 4.6, df = 20,* P* < 0.001).

**Figure 2:**
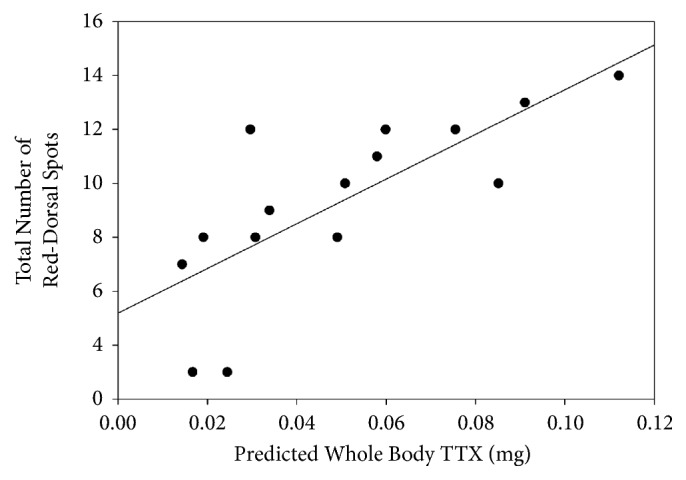
Regression of the total number of red dorsal spots and the predicted whole body TTX (mg) of Eastern newt (*Notophthalmus viridescens*) efts. Efts with more red dorsal spots possess greater concentrations of TTX in the skin (F = 17.5, R^2^ = 0.57, P = 0.001). The whole body TTX estimate for each eft was the average of the paired estimates from the punch containing and lacking a red dorsal spot.

Newt efts possessed almost a tenfold difference in whole newt TTX levels, ranging between 0.014 and 0.11 mg (mean ± SD; 0.05±0.03). The whole newt toxicity for adults was lower, ranging between 0.005 and 0.057 mg (mean ± SD; 0.035±0.017). A t-test found that adult newts possess significantly less TTX than efts (*t* = 4.6, df = 20,* P* < 0.001, Figure 1). A paired t-test found no significant difference between the concentration of TTX in the red spots versus areas lacking these spots in adult newts (*t* = -0.453, df = 6,* P* = 0.67, Figure 1) or in efts (*t* = 0.014, df = 14,* P* = 0.99, Figure 1). A linear regression detected a significant positive relationship between the total toxicity of an eft and the number of red dorsal spots (F = 17.5, R^2^ = 0.57, P = 0.001, Figure 2). These data indicate that as the number of red dorsal spots increases, total toxicity of the eft increases.

## 4. Discussion

Our study shows that the red dorsal spots do not contain elevated TTX concentrations relative to areas lacking these spots in eft nor adult Eastern newts. There is extreme variability in the number of red spots in this species [16, 24], with efts in our study possessing between 3 and 14 spots. The relationship between toxicity and the number of aposematic spots indicates that there could be a fitness advantage to possessing more spots. Several predators, including invertebrates, salamanders, some frogs, most reptiles, and birds, are deterred from eating Eastern newts due to the presence of TTX [11, 14, 15, 25, 26]. Others, such as turtles, bullfrogs, and raccoons, have been documented to consume adult or eft newts [11, 27, 28]. In the species that are capable of consuming newts, the red spots could serve as an indicator of the toxicity of their prey. While the red dorsal spots are small in comparison to the bright orange dorsal pigmentation of the entire eft, fine-scale pattern discrimination has been documented in species that consume unpalatable prey. For example, rufous-tailed jacamars (*Galbula ruficauda*) are able to discriminate between minute differences on the wing patterning of* Heliconius* butterflies, leading to attacks on novel phenotypes [29]. While the number of red spots on the dorsum is independent of sex, the spots of adult male newts are brighter and have a redder hue than those of females [16]. In addition, larger males have brighter spots than smaller individuals [16]. These results suggest a sexually selected function for the red spots in adults, whereby females could gain information about mate quality (including toxicity). Nevertheless, the relationship between toxicity, spotting, and its role in mate selection deserves further investigation.

Another interesting finding is that newt efts contained higher concentrations of TTX (per plug) relative to the aquatic adults. Previous investigations into the toxicity level of these life-history stages has yielded mixed results. For example, a study by Yotsu-Yamashita and Mebs [12] investigated the levels of TTX, 6-epiTTX, and 11-oxoTTX between eft and adult newts. While efts contained higher concentrations of 6-epiTTX, levels of both TTX and 11-oxoTTX were not significantly different [12]. Our results contradict those of Yotsu-Yamashita and Mebs [12] and Yotsu-Yamashita et al. [13] and indicate efts and adults do differ in the concentration of TTX. In particular, a single plug from newt efts from our study had 517±254 ng TTX (mean ± SD) whereas the plugs from the adults contained only 74±41 ng TTX. This roughly 7 times higher toxicity in efts relative to adults from Virginia is also supported by Brodie [11] and Brodie et al. [9]. These authors found that, in newts from North Carolina, eft skin extract was ten times more lethal to white mice relative to adult skin extract. Recently, Yotsu-Yamashita et al. [13] conducted a study on the toxicity of newts from a large geographic region of the eastern United States. While the authors claim the toxicity of efts and adults was similar (our results contradict this claim for one of the populations included in their study), no data were presented and the authors appear to have combined all eft and adult values together for each population. If eft and adults do differ in TTX levels, lumping them together could have dramatically skewed any geographic differences between populations. This is especially true if the ratio of efts to adults they sampled was different for different populations.

The results of this study indicate that the red spots on the dorsum of eft and adult newts do not contain elevated TTX levels relative to patches of skin that lack these bright spots. Nevertheless, the red spots could serve as an indicator of toxicity given the positive relationship between the number of red spots and whole newt toxicity. Finally, efts from Virginia possessed greater concentrations of TTX in their skin relative to the aquatic adults.

## Data Availability

The data used to support the findings of this study are available from the corresponding author upon request.
